# A diagnostic algorithm combining clinical and molecular data distinguishes Kawasaki disease from other febrile illnesses

**DOI:** 10.1186/1741-7015-9-130

**Published:** 2011-12-06

**Authors:** Xuefeng B Ling, Kenneth Lau, John T Kanegaye, Zheng Pan, Sihua Peng, Jun Ji, Gigi Liu, Yuichiro Sato, Tom TS Yu, John C Whitin, James Schilling, Jane C Burns, Harvey J Cohen

**Affiliations:** 1 Department of Pediatrics, Stanford University, Stanford, CA 94305, USA; 2 Department of Pediatrics, University of California San Diego, La Jolla, CA 92093, USA; 3 Rady Children's Hospital San Diego, San Diego, CA 92123, USA; 4 Amgen Inc, South San Francisco, CA 94080, USA

## Abstract

**Background:**

Kawasaki disease is an acute vasculitis of infants and young children that is recognized through a constellation of clinical signs that can mimic other benign conditions of childhood. The etiology remains unknown and there is no specific laboratory-based test to identify patients with Kawasaki disease. Treatment to prevent the complication of coronary artery aneurysms is most effective if administered early in the course of the illness. We sought to develop a diagnostic algorithm to help clinicians distinguish Kawasaki disease patients from febrile controls to allow timely initiation of treatment.

**Methods:**

Urine peptidome profiling and whole blood cell type-specific gene expression analyses were integrated with clinical multivariate analysis to improve differentiation of Kawasaki disease subjects from febrile controls.

**Results:**

Comparative analyses of multidimensional protein identification using 23 pooled Kawasaki disease and 23 pooled febrile control urine peptide samples revealed 139 candidate markers, of which 13 were confirmed (area under the receiver operating characteristic curve (ROC AUC 0.919)) in an independent cohort of 30 Kawasaki disease and 30 febrile control urine peptidomes. Cell type-specific analysis of microarrays (csSAM) on 26 Kawasaki disease and 13 febrile control whole blood samples revealed a 32-lymphocyte-specific-gene panel (ROC AUC 0.969). The integration of the urine/blood based biomarker panels and a multivariate analysis of 7 clinical parameters (ROC AUC 0.803) effectively stratified 441 Kawasaki disease and 342 febrile control subjects to diagnose Kawasaki disease.

**Conclusions:**

A hybrid approach using a multi-step diagnostic algorithm integrating both clinical and molecular findings was successful in differentiating children with acute Kawasaki disease from febrile controls.

## Background

Kawasaki disease (KD) is an acute vasculitis that affects infants and children and is the leading cause of acquired pediatric heart disease in the U.S. and Japan [1]. The cause of KD remains unknown, although epidemiologic and clinical observations suggest that an infectious agent(s) may trigger the inflammatory process in genetically susceptible hosts, who then manifest the clinical syndrome [2]. The diagnosis of KD is currently based on clinical signs and supportive non-specific laboratory testing [3,4]. There is no specific diagnostic test for the disease. If not diagnosed and treated promptly, patients with KD may develop coronary artery dilatation or aneurysms. The cardiovascular damage can be largely prevented by timely administration of intravenous immunoglobulin (IVIG). Thus, there is an acute need for a sensitive and specific diagnostic test or panel that can facilitate diagnosis and permit timely treatment.

We postulated that specific patterns of blood leukocyte gene expression and plasma or urine protein excretion patterns are associated with KD. Identification of these biomarkers could provide insight into the pathophysiology of KD, and even give clues to its etiology. Investigators have taken both genomic and proteomic approaches to biomarker discovery in KD. Transcriptional profiling of blood leukocytes has identified disease-specific expression patterns [5-9]. Protein biomarker studies [7,10-14] have revealed elevated levels of cytokines, chemokines, and acute phase reactants, but none are uniquely elevated in KD.

Our previous analysis [8,9] of peripheral whole blood gene expression compared acute KD and febrile control (FC) patients, revealing increased relative abundance of transcripts associated with innate immune and proinflammatory responses and decreased abundance of transcripts associated with natural killer cells and CD8+ lymphocytes. Expression analyses of separate blood cell type would be more informative. However, the isolation of peripheral blood subsets is cumbersome and may alter gene expression. We, therefore, compared KD and FC whole blood gene expression using cell type-specific significance analysis of microarrays (csSAM [15]) to analyze differential gene expression for each cell type in a biological sample based on microarray data and relative cell-type frequencies.

Urine is a rich source of proteolytically cleaved proteins cleared from plasma by the kidneys. Profiling analysis of the urinary proteome/peptidome is highly informative for both uro-genital and systemic disease classification [16,17]. Using this approach, we previously described urine peptide biomarkers associated with renal transplantation rejection [18,19] and systemic juvenile idiopathic arthritis (SJIA)[20]. We, therefore, performed mass spectrometric analyses of urinary peptides in KD and FC patients.

In this study, we applied an ensemble data-mining approach [21] integrating either blood cell type-specific gene expression or urine peptidome profiling with clinical multivariate analysis to improve the diagnosis of KD.

## Materials and methods

### Patient demographics and samples

Informed consent was obtained from the parents of all subjects and assent from all subjects > 6 years of age. This study was approved by the human subjects protection programs at the University of California San Diego (UCSD) and Stanford University. Inclusion criteria for KD subjects were based on the American Heart Association Guidelines [22]. All KD subjects had fever for at least three days and four of five classic criteria or three or fewer criteria with coronary artery abnormalities documented by echocardiogram. The 441 KD patients were distributed according to either the IVIG therapy outcome (Non responder: n = 68; Responder: n = 271; Late treatment: n = 55; Non treated: n = 16; IVIG + Remicade for coronary artery aneurysms: n = 10; data not available: n = 21) or the coronary artery lesion status (Normal: n = 323; Aneurysms: n = 34; Dilated: n = 83; Data not available: n = 1). FC subjects were age-similar children evaluated for fever accompanied by at least one of the KD criteria (rash, conjunctival injection, oral mucosa changes, extremity changes, enlarged cervical lymph node). Febrile children with prominent respiratory or gastrointestinal symptoms were specifically excluded such that the majority of the controls had KD in the differential diagnosis of their condition. All subjects provided samples of blood and urine and underwent other diagnostic tests at the discretion of the managing clinicians. De-identified clinical laboratory test data were extracted from the UCSD KD electronic database for multivariate analysis. FC patients had a clinically or culture proven etiology for their febrile illnesses or underwent resolution of fever and clinical signs within three days of obtaining their clinical samples (designated as 'viral syndrome').

We compiled 3 cohorts of KD and FC subjects evaluated for their febrile illnesses at Rady Children's Hospital San Diego (Tables 1, 2 and 3): 783 for clinical score development (clinical group: 441 KD and 342 FC); 106 for urine peptidome analysis (urine group: 53 KD and 53 FC); and 39 for cell type-specific microarray analysis of whole blood (blood group: 23 KD and 16 FC). The blood group (KD n = 23, FC n = 16) is a subset of previously analyzed samples (NCBI GEO GSE15297 [9], peripheral whole blood expression analysis) with complete clinical data for all subjects. We chose KD and FC patients for the urine and blood groups with similar age and same gender. Patient demographic data were analyzed using SAS 9.2 (SAS Institute Inc., Cary, NC, USA). The KD patients in the clinical group were more predominantly male and were younger than the FC patients but did not differ in ethnicity (Table 1). The 53 KD and 53 FC patients in the urine group were age-matched and did not differ in gender or ethnicity. Asian ethnicity was more common among KD subjects in the blood group.

**Table 1:** Demographics of the 783 Patients with Kawasaki Disease or Febrile Conditions

	Kawasaki Disease	Febrile Condition	*P*-value
	
	n = 441 (56.3%)	n = 342 (43.7%)	
Age (months)^a^	35.6 [1.0, 178.0, 29.7-33.9]	44.0 [1.0, 210.0, 39.0-45.3]	0.002
Male	273 (61.9%)	190 (55.6%)	0.002
Ethnicity			
Asian	59 (13.5%)	31 (9.1%)	0.116
African American	17 (3.9%)	12 (3.5%)	
Caucasian	117 (26.7%)	87 (25.5%)	
Hispanic	149 (34.0%)	129 (37.8%)	
Mixed	90 (20.6%)	69 (20.2%)	

^a^Reported as means with minimum, maximum, and 95% confidence interval in bracket. T-test was used.

All other variables were reported as the number of patients and were analyzed using Fisher's Exact test.

**Table 2:** Demographics of the 106 Patients with Urine Peptidome Data

	Kawasaki Disease	Febrile Condition	*P*-value
	
	n = 53 (5.0%)	n = 53 (50.0%)	
Age (months)^a^	50.6 [3.0, 182.0, 33.6-49.8]	54.2 [5.0, 209.0, 39.9-58.8]	0.670
Male	36 (67.9%)	33 (62.3%)	0.409
Ethnicity			
Asian	7 (13.4%)	6 (11.3%)	0.095
African American	1 (1.9%)	1 (1.9%)	
Caucasian	10 (19.2%)	12 (22.6%)	
Hispanic	18 (34.6%)	16 (30.2%)	
Mixed	16 (30.8%)	11 (20.8%)	

^a^Reported as means with minimum, maximum, and 95% confidence interval in bracket. T-test was used.

All other variables were reported as the number of patients and were analyzed using Fisher's Exact test.

**Table 3:** Demographics of the 39 Patients with Microarray Data

	Kawasaki Disease	Febrile Condition	*P*-value
	
	n = 23 (59.0%)	n = 16 (41.0%)	
Age (months)^a^	43.2 [5.0, 152.0, 27.1-49.6]	64.3 [6.0, 188.0, 38.1-90.6]	0.125
Male	15 (65.2%)	9 (69.2%)	1.000
Ethnicity			
Asian	8 (34.8%)	0 (0%)	0.044
African American	1 (4.4%)	0 (0%)	
Caucasian	5 (21.7%)	7 (43.8%)	
Hispanic	6 (26.1%)	8 (50.0%)	
Mixed	3 (13.0%)	1 (6.3%)	

^a^Reported as means with minimum, maximum, and 95% confidence interval in bracket. T-test was used.

All other variables were reported as the number of patients and were analyzed using Fisher's Exact test.

### KD clinical score calculation

**Figure 1:**
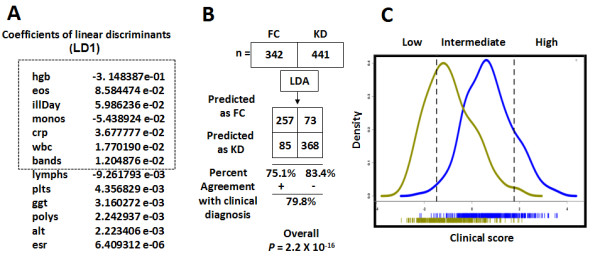
**Linear discriminant analysis of KD training cohort patients**. **(A) **The calculated coefficients of linear discriminants (LD1) of each assayed clinical parameter. **(B) **A modified 2 × 2 contingency table depicting the agreement of classifications with clinical diagnosis. **(C) **Density plots of the clinical score distribution of both the KD and FC patients. KD, blue density plot; FC, yellow density plot. We stratified all the KD and FC patients, using the clinical scores, into low, intermediate and high groups, where the group boundaries were decided by the diagnosis with 95% accuracy (two dotted vertical lines). Abbreviations: FC, febrile controls; KD, Kawasaki disease.

We used linear discriminant analysis (LDA) to stratify individual subjects based on a series of clinical exploratory variables. R http://www.r-project.org/ library MASS function 'lda' was utilized. Coefficients of linear discriminants (LD1) were calculated as a measure of the association of each variable with the final diagnosis. The discriminant score was calculated from the seven variables (Figure 1) with the largest (absolute value) coefficients. All patients were stratified into subgroups with low (5% likelihood KD), intermediate, and high (95% likelihood KD) clinical scores.

### Microarray analysis of peripheral whole blood

We performed csSAM [15] to analyze differential gene expression for each blood cell type in our previous KD array data set (NCBI GEO GSE15297 [9]). Our expression analysis de-convoluted the major blood cell types: lymphocytes, neutrophils, immature neutrophils (band forms), monocytes, and eosinophils. For each gene, in each cell type, we calculated the contrast in its de-convoluted expression between KD and FC groups. The false discovery rate (FDR) was calculated as the ratio of genes whose differentiation exceeded a given threshold in the real dataset compared with the number of genes found significant by multiple permutations of the samples.

### Urine collection, storage and processing

Urine samples (5-10 mL) were either spontaneously voided or collected by bladder catheterization and held at 4°C for up to 48 hours before centrifugation (2,000 g × 20 minutes at room temperature) and freezing of the supernatant at -70°C. The details of urine processing, preparation of peptides, extraction and fractionation are reported elsewhere [17].

### Urine peptidomic data analysis

We pooled equal peptide content from 23 KD and 23 FC (Additional file 1, Supplementary Table 3) and subjected the pooled peptidome samples to multi-dimensional protein identification technology (MUDPIT: strong cation exchange (SCX) and reverse phase (RP) separations) analysis using Fourier transform ion cyclotron resonance (FT ICR) mass spectrometry. The mass spectrometer's data-dependent acquisition isolates peptides as they elute and subjects them to Collision-Induced Dissociation, recording the fragment ions in a tandem mass spectrum. These spectra are matched to database peptide sequences by searching MS/MS (Mass spectrometry/Mass spectrometry) spectra against the Swiss-Prot database (version, June 10, 2008) restricted to human entries (15,720 sequences) using the SEQUEST search engine. Searches were restricted to 50 and 100 ppm for parent and fragment ions, respectively. No enzyme restriction was selected. Since we were focusing on naturally occurring peptides, matches were considered significant when they were above the statistically significant threshold (as returned by SEQUEST BioWorks™ rev.3.3.1 SP1). Different fragmentation techniques were used for the validation of a peptide sequence, as well as for the detection, localization and characterization of the post-translational modifications. Due to the strong correlation between relative protein/peptide abundance and spectral counting summing all MS/MS spectra observed for the same peptide, the spectral counting method was used to compare the peptide abundance between KD and FC pooled samples. If the spectral counting of a peptide differed by two between KD and FC pooled samples, this peptide was chosen for ABI5800 matrix-assisted laser desorption/ionization (MALDI) TOF (Time of Flight) confirmation analysis. The individual peptidomes of 30 KD and 30 FC subjects (Additional file 1, Supplementary Table 4) were subjected to liquid chromatography-mass spectrometry (LCMS) based urine peptide profiling by ABI 5800. We targeted the 139 peptide biomarker candidates revealed by MUDPIT analysis and used their mass to charge ratio (m/z) values of the ions across all the LC fractions detected to construct extracted ion chromatograms (XICs) of individual urine samples. Windows for XIC construction were 25 ppm for m/z. Peak intensity values were normalized to the mean intensity of all peaks within a sample and then to the mean of the individual peptide ions across the samples. To follow up the potential peptide biomarkers, the statistical significance of each peptide's peak intensity between KD and FC groups was analyzed using the Mann Whitney U test and Student's t test. The urine peptide biomarker panel was analyzed by supporting vector machine (SVM) algorithm (R e1071 package). ROC analysis was performed [23,24] to evaluate the performance of the clinical and molecular-based classifiers in the diagnosis of KD. Area under the ROC curve was calculated using RORC package [24].

### Sequential predictive analysis integrating clinical and molecular findings for KD diagnosis

To improve the diagnosis of patients with the intermediate clinical scores, we used Ensemble Data Mining Methods, also known as Committee Methods or Model Combiners [21], to combine the clinical and molecular biomarker classifiers in order to derive practical algorithms for KD management. These machine learning methods combine the advantages of multiple models to achieve better predictive accuracy than is possible with any individual model [21]. We first stratified subjects into low, intermediate, and high risk groups based on clinical scores. Patients with intermediate KD clinical scores were further analyzed by either blood lymphocyte expression based or by urine peptidome based classifiers to improve diagnostic sensitivity and specificity.

### Biological pathway analysis

Biological pathway analysis was performed with the Ingenuity IPA system (Ingenuity Systems, Redwood City, CA). To identify the canonical pathways that encompassed our KD biomarkers, 87 genes (94 significant probes) revealed by the cell type-specific gene expression studies of peripheral whole blood samples, and 13 significant urine peptide markers were mapped to known entries in the IPA canonical pathway database. The significance of the pathway was tested using Bioconductor http://www.bioconductor.org packages as previously described [25] and pathways with *P *value < 0.05 were chosen for further analysis.

## Results

### Development of KD clinical score

A data set of 783 patients, 342 FC and 441 KD, had complete records for 13 clinical and laboratory observations, which were used for exploratory multivariate linear discriminant analysis (LDA) (Tables 1, 2 and 3): number of days of fever at time of clinical visit (illDay), total white blood cell (wbc), percentage monocytes (monos), lymphocytes (lymphs), eosinophils (eos), neutrophils (polys), immature neutrophils (bands), platelet counts (plts), hemoglobin (hgb), C-reactive protein (crp), gamma-glutamyl transferase (ggt), alanine aminotransferase (alt), and erythrocyte sedimentation rate (ESR). LDA created linear combinations of these clinical variables and calculated coefficients LD1 to optimize separation between KD and FC groups (Figure 1A). The discriminant model predicts clinical diagnosis with 79.8% overall accuracy (Figure 1B, Fisher exact test *P *= 2.2 × 10^-16^). The seven variables with the largest absolute values of coefficients LD1 were: days of illness, concentrations of hemoglobin and C-reactive protein, white blood cell count, and percentages of eosinophils, monocytes, and immature neutrophils (Figure 1A) for discriminant score calculation. The LDA discriminant scoring metric, designated as the KD 'clinical score', enables the seven clinical variables to be collectively interpreted on a scale, rather than a strict binary discrimination. Histograms of KD clinical scores demonstrate the distribution and considerable overlap of KD and FC patients (Figure 1C). Patients were stratified into three levels of risk for KD, determined by 95% correct classification effectiveness: low (clinical score < -1.48; 108 (96%) FC, 5 (4%) KD), intermediate (-1.481 ≤ clinical score ≤ 1.775, 366 KD and 230 FC) and high (clinical score > 1.775; 70 (95%) KD, 4 (5%) FC) groups. Although the clinical score was accurate for subjects in the low (n = 113) and high (n = 74) clinical scoring groups, 596 patients (76%) had intermediate scores and remained unassigned by our clinical scoring algorithm.

### Cell type-specific significance analysis (csSAM) of peripheral whole blood expression to differentiate KD and FC patients

**Figure 2:**
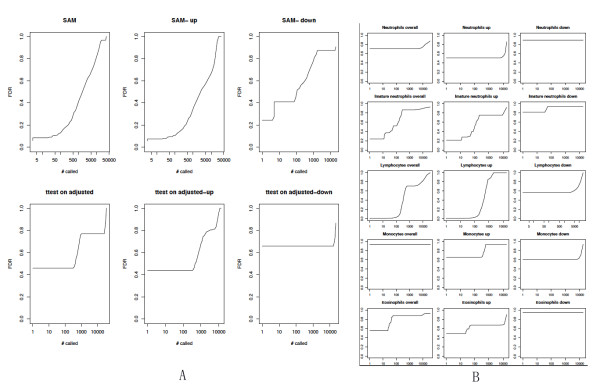
**Cell type-specific significance analysis of KD and FC whole blood microarray data**. (Microarray analysis of peripheral whole blood, FC n = 16, KD n = 23, Testing data set 1). **(A**) SAM analysis revealing no differentially expressed genes in whole blood. **(B) **csSAM revealing differential expression in lymphocytes, but not in other cell types. Up: up-regulated in FC. Down: down-regulated in FC. Y axis: false discovery rate (FDR); X axis: number of differential genes at a given FDR. Microarray analysis of peripheral whole blood, FC n = 16, KD n = 23 (Testing data set 1). Abbreviations: FC, febrile controls; KD, Kawasaki disease; SAM, significance analysis of microarrays; scSAM, cell-type specific significance analysis of microarrays.

We employed the recently developed csSAM [15] method, combining our KD array data set (NCBI GEO GSE15297 [9], blood testing cohort) and patients' relative cell type frequencies to analyze differential gene expression for each blood cell type in KD (n = 23) and FC (n = 16) subjects' whole blood. Whole-blood differential expression analysis using the Significance Analysis of Microarray (SAM) algorithm [26], revealed no differentially expressed genes between the KD and FC groups at a relatively permissive FDR of 0.1 (Figure 2A). For each of the KD and FC patients, we de-convoluted the cell type-specific gene expression profile, using csSAM, to perform cell type-specific differential expression analysis. Although the whole blood SAM analysis revealed no significant differentially expressed genes, and the lymphocyte count itself did not contribute to the clinical score, the csSAM analysis identified 87 differentially (down regulated in KD) expressed genes (94 gene probes; Additional file 1, Supplementary Table 1) in lymphocytes. Although eosinophil, monocyte and immature neutrophil relative counts had large LD1 coefficients for the KD clinical score, csSAM analysis identified no marker genes (FDR < 0.05) for these blood cell types (Figure 2B). Additional file 1, Supplementary Figure 1 summarizes the training, 10 fold cross-validation, and test errors for different values of the threshold, revealing an effective KD/FC diagnostic gene marker panel (32 unique genes; top 36 gene probes in Additional file 1, Supplementary Table 1).

### Urine peptidome analysis discriminating KD and FC patients

**Figure 3:**
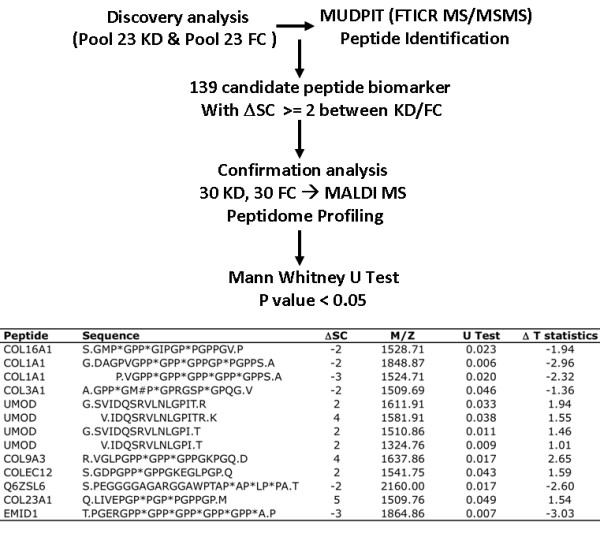
**Urine peptidome analysis**. Top panel summarizes the MUDPIT discovery and MALDI MS confirmation processes. Bottom table lists the 13 confirmed urine peptide biomarker discriminating KD and FC. M/Z: mass to charge ratio. ΔSC: spectral counting difference. Post translation modifications: * hydroxylation; # methionine oxidation. U Test: *P *value. Abbreviations: FC, febrile controls; KD, Kawasaki disease; MALDI MS, Matrix-assisted laser desorption/ionization (MALDI) mass spectrometry; MUDPIT, multi-dimensional protein identification technology.

As shown in Figure 3, for urine peptidome analyses, we have employed a combination of methods of multi-dimensional protein identification technology (MUDPIT: strong cation exchange SCX and reverse phase RP separations) analysis using Fourier transform ion cyclotron resonance (FT ICR) to discover candidate biomarkers in pooled KD (n = 23) and FC (n = 23) urine discovering cohort samples. Matrix-assisted laser desorption/ionization (MALDI) mass spectrometric (MS) TOF analysis was used to confirm these biomarkers in individual KD (n = 30) and FC (n = 30) urine testing cohort samples. Our exploratory MUDPIT analysis of pooled urine peptidomes yielded 139 candidate peptide biomarkers (Figure 3). Subsequent MALDI TOF analysis confirmed the statistical significance of 13 urine peptides (Figure 3), which are derived from 9 protein precursors (collagen type 1 6 alpha 1, collagen type 1 alpha 1, collagen type 3 alpha 1, uromodulin, collagen type 9 alpha 3, collagen type 23 alpha 1, collectin sub-family member 12, unnamed protein product Q6ZSL6, and EMI domain containing 1). Sequence alignment of these peptides revealed tight sequence clusters for the two COL1A1 and four UMOD peptides.

### A novel KD diagnostic algorithm integrating clinical and molecular biomarker findings

**Figure 4:**
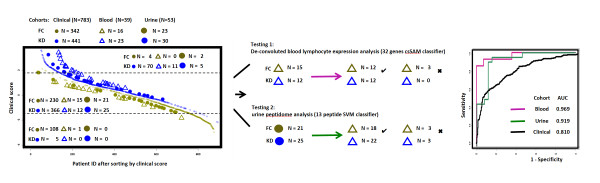
**A sequential predictive algorithm integrating clinical and molecular biomarker findings to improve KD diagnosis**. KD, blue; FC yellow. Left panel: clinical, blood testing, and urine testing cohorts were stratified and ordered according to their clinical scores. Patients with intermediate scores were further analyzed by 32 gene or 13 urine peptide based classifiers to discriminate KD and FC. Right panel: ROC analysis of all three-cohort patients with intermediate clinical scores analyzed by either clinical score (black), cell-specific, whole blood gene-based (red) and urine peptide-based (green) classifiers. Abbreviations: FC, febrile controls; KD, Kawasaki disease; ROC, receiver operating characteristic.

We first computed KD clinical scores for all patients in the clinical training, blood testing, and urine testing cohorts (Figure 4, left panel). Although the clinical score had high sensitivity (blood: 11 of 11; urine: 5 of 5) and specificity (blood: 1 of 1; urine: 0 of 2) when limited to the low and high clinical score groups, the majority of the blood (27 of 39) and urine (46 of 53) testing cohorts were in the intermediate group where FC and KD patients had considerable clinical overlap.

We applied gene expression or urine peptide based classifiers to better discriminate KD from FC subjects with intermediate KD clinical scores (Figure 4, middle panel). The 32-lymphocyte-specific-gene-marker panel correctly classified 12 of 15 FC and 12 of 12 KD blood group patients with intermediate clinical scores. The 13-urine-peptide-biomarker panel correctly classified 18 of 21 FC and 22 of 25 KD urine group patients with intermediate clinical scores. ROC (Figure 4, right panel) analysis revealed that molecular analyses of blood cell-specific gene expression (AUC 0.969) and of the urine peptidome (AUC 0.919) were superior to the clinical score (AUC 0.810) in differentiating KD from FC patients with intermediate clinical scores. This analysis suggests that the integration of clinical and molecular based panels provides an effective strategy for KD diagnosis. Febrile patients with low and high KD clinical scores are diagnosed with 95% confidence and need no further evaluation. Additional molecular-based testing, by either blood array profiling or urine peptidome analysis, refines the diagnostic performance for the remaining patients with intermediate clinical scores.

### Biological pathway analyses of blood lymphocyte-specific gene markers and urine peptide biomarkers

**Figure 5:**
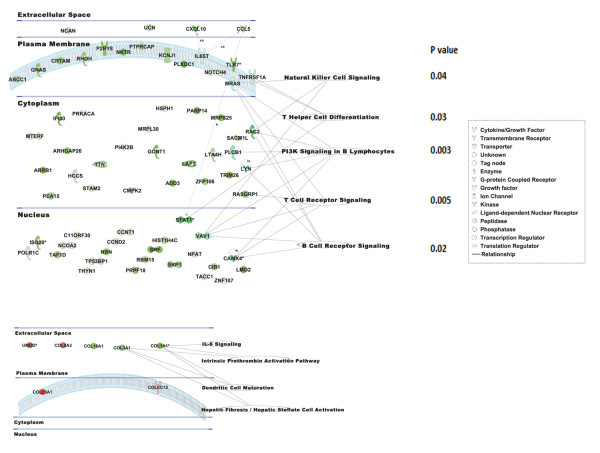
**Pathway analysis of the lymphocte-specific gene (A) or urine peptide (B) markers**. Data mining software (Ingenuity Systems, http://www.ingenuity.com, Redwood City, CA) was used with differentially (KD vs FC) expressed genes or peptides to identify gene ontology groups and relevant canonical signaling pathways. The intensity of the node color indicates the degree of up- (red) or down- (green) regulation in KD. Nodes are displayed using shapes that represent the functional classes of the gene products, and different line types represent various relationships. Relationships are primarily due to co-expression. Abbreviations: FC, febrile controls; KD, Kawasaki disease.

To characterize the canonical pathways in which our KD biomarkers are involved, 87 lymphocyte gene markers (94 significant probes) revealed by the cell type-specific expression of peripheral whole blood samples, and 13 confirmed urine peptide markers were mapped to known entries in the IPA (Ingenuity Pathway Analysis) canonical pathway database (Figure 5). Cellular location analysis revealed that > 70% of the significant gene products reside within the cytoplasm and nucleus. In contrast, as expected, all of the significant urine peptides are derived from proteins located either in the extracellular space or on the plasma membrane. Pathway significance analysis [25] of blood lymphocyte-specific gene markers revealed that PI3K signaling (*P *= 0.003), T cell receptor signaling (*P *= 0.005), B cell receptor signaling (*P *= 0.02), T helper cell differentiation (*P *= 0.03) and natural killer cell signaling (*P *= 0.04) were significantly down-regulated in KD compared to FC patients. Urine peptidome pathway analysis revealed that the intrinsic prothrombin activation pathway (*P *= 3.04 × 10^-5^), hepatic fibrosis/hepatic stellate cell activation (*P *= 6.49 × 10^-4^), dendritic cell maturation (*P *= 0.001), and IL-6 signaling (*P *= 0.01) were significantly down-regulated in KD compared to FC.

## Discussion

We have identified three different biomarker panels (7 clinical parameters, 32 blood lymphocyte-specific genes, 13 urine peptides) and developed an integrated algorithm to accurately diagnose KD.

The clinical data we used in the multivariate analysis are routinely obtained during the evaluation of fever. However, clinicians have not used scoring systems derived by multivariate techniques for KD diagnosis. Although the clinical score correctly classified only 80% of febrile patients, patients with either low or high KD clinical scores were diagnosed as FC or KD respectively with 95% accuracy. For febrile patients with the confident diagnosis of KD, timely administration of IVIG can thus be feasible to prevent the development of coronary artery dilatation or aneurysms. For febrile patients with intermediate clinical scores for whom confident diagnosis is not feasible, we developed a sequential algorithm, integrating clinical and molecular findings to improve KD diagnosis. Both the peripheral blood cell type-specific analysis and the urine peptidome biomarker analysis yielded sensitive and specific classifiers, which performed well in the diagnosis of KD. Prospective testing of these biomarker panels will be necessary to confirm their diagnostic utilities.

The csSAM-derived lymphocyte-specific gene markers and their mapped canonical pathways, for example PI3K signaling in B cells and T cell receptor signaling, provide insight into the host response in KD. Confirmation of our de-convolution observations on independent samples will establish the role of these genes as KD biomarkers. Validation of these markers may help to focus the search for the etiology of KD on agents that suppress specific lymphocyte gene expression.

The overlapping sequences of the two COL1A1 and four UMOD peptides suggests that these peptide biomarkers reflect differential activities of disease-related proteases or their inhibitors such as TIMP1 or matrix metalloproteinases in KD [6,27-32]. Serum peptide biomarker analysis of cancer subjects [33] has demonstrated overlapping peptide biomarkers generated by disease-specific exo-peptidase activity. We have also observed tight clusters of urine peptide biomarkers in renal allograft dysfunction [19] and SJIA [19]. Therefore, the discovery of multiple overlapping collagen and uromodulin peptides suggests that the pathophysiology of KD involves the active degradation of proteins including collagen and uromodulin.

With respect to the concern regarding incomplete KD cases hidden among the FC, we agree that inaccurate diagnosis is always one of the limitations in the absence of a gold standard diagnostic test. However, FC in this study included only patients whose illness resolved within three days of blood sampling OR for whom a definite diagnosis was established (for example osteomyelitis, JIA). None of the FC included here had peeling in the convalescent phase. As for the KD patients, we have maintained a stable rate of coronary artery aneurysms from year to year (approximately 9%) suggesting that our diagnostic practices are stable. All the KD patients in this study were evaluated by one of two experienced clinicians at a single medical center. In this study, most of the FCs were enrolled by our team member, thus assuring consistency in diagnosis and sample collection. Our study is unique in focusing on a clinically relevant control group of children with fever who were actually being evaluated to rule in or rule out KD. All FC were evaluated with a standardized set of clinical laboratory tests that was also used to evaluate our KD patients. Our study also differs from many previous investigations on KD that used samples collected from a large number of hospitals that cared for only a few KD patients each. Therefore, a big problem with consistency in these studies was expected for comparative studies between KD and FC.

Although all FC subjects in this study had laboratory testing for KD as recommended by the American Heart Association (AHA), very few FC had echocardiographic studies done. This is indeed a limitation. Although we acknowledge the potential inaccurate diagnosis of incomplete KD, our status as the sole freestanding children's hospital, sole KD referral center, and sole pediatric emergency department in San Diego County (catchment area of 5 million people) maximizes the likelihood that FC with persistent or progressive illness confused with KD would be captured during a return visit.

We recognize several limitations to the current molecular study for future translations of these biomarkers into bedside practice. First, the small sample sizes limit the power of our biomarker analyses to validate statistically significant associations and to avoid spurious discovery. Future prospective studies with larger sample sizes will be needed to validate our cell type-specific gene expression and urine peptide biomarkers. A second limitation of our study was the lack of formal assessment by clinicians of the pre-test probability of KD in the subjects included in this study. While a large proportion of the febrile controls were referred to our emergency department by physicians for evaluation of possible KD, this was not uniformly true as some febrile controls likely had a low pre-test probability of KD. Since the pre-test probability is an important consideration in evaluating the performance of a diagnostic test, collection of this information will be critical in the next testing phase of a KD diagnostic test. Third, the application of both the cell-specific transcript patterns and urine peptide biomarkers for the diagnosis of KD will require development of technology for the rapid identification of both whole blood transcripts and urine protein fragments in the clinical laboratory.

Our flexible clinical scoring metric is amenable to automation to develop data-driven predictive systems. Consistent with the current mandate to improve electronic medical record (EMR) use [34] and future interoperability between the hospital EMR and our predictive algorithm based applications consisting of demographic, clinical and genomic/proteomic data can serve an effective platform to allow interfacing between interdisciplinary teams (bed and bench side; what is known and what is practiced) for productive translational medicine.

## Conclusion

To the best of our knowledge, this is the first report describing a method integrating both clinical and molecular findings to discriminate KD from FC. Subsequent testing feedback from prospective KD/FC EMR data can be expected to further refine the clinical scoring metric and improve the KD diagnosis [34].

## Abbreviations

EMR: electronic medical record; MUDPIT: multidimensional protein identification; csSAM: cell type-specific significance analysis of microarrays; LDA: linear discriminant analysis; SVM: supporting vector machine; ROC: receiver operating characteristic; AUC: area under the curve; KD: Kawasaki disease; FC: febrile control; MALDI: Matrix-assisted laser desorption/ionization; FTICR: Fourier transform ion cyclotron resonance; MS: mass spectrometry; SC: spectral counting.

## Competing interests

The authors declare that they have no competing interests.

## Authors' contributions

XBL and HJC are the major contributors responsible for data analysis and project management. XBL, HJC, JCB and JS contributed to overall experimental design and assay platform setup. ZP, SP and JJ contributed to the pathway data analysis. GGL and YS contributed to the patient demographic analysis. KL contributed to the urine peptidome profiling analysis. KL, TTSY and JCW contributed to the sample storage and process. XBL, JTK, JCB and HJC contributed to the manuscript writing. All authors read and approved the final manuscript.

## Pre-publication history

The pre-publication history for this paper can be accessed here:

http://www.biomedcentral.com/1741-7015/9/130/prepub

## Supplementary Material

Additional file 1 **Supplementary Figure 1, Supplementary Figure 2; Supplementary Table 1, Supplementary Table 2, Supplementary Table 3, and Supplementary Table 4**. Click here for file
