# Supplementary material for: A diagnostic algorithm combining clinical and molecular data distinguishes Kawasaki disease from other febrile illnesses
Source: BMC Med. 2011 Dec 6;9:130. doi: 10.1186/1741-7015-9-130 (PMC3251532; doi:10.1186/1741-7015-9-130)
Supplement: Additional file 1 — Supplementary Figure 1, Supplementary Figure 2; Supplementary Table 1, Supplementary Table 2, Supplementary Table 3, and Supplementary Table 4. [file 1741-7015-9-130-S1.PDF]

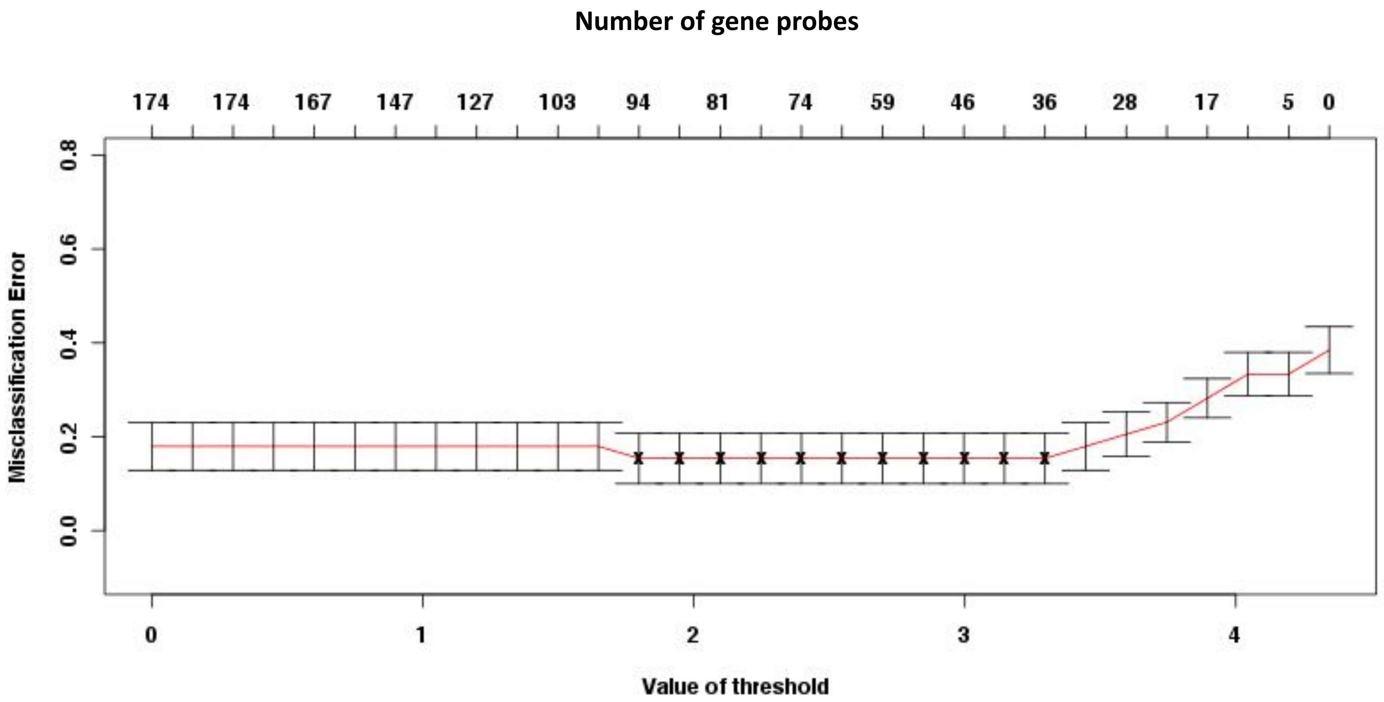

Supplementary Figure 1

Supplementary Figure 2.

Density plot analysis of the Z score (LAD and RCA) to quantify coronary artery lesions in KD patients.

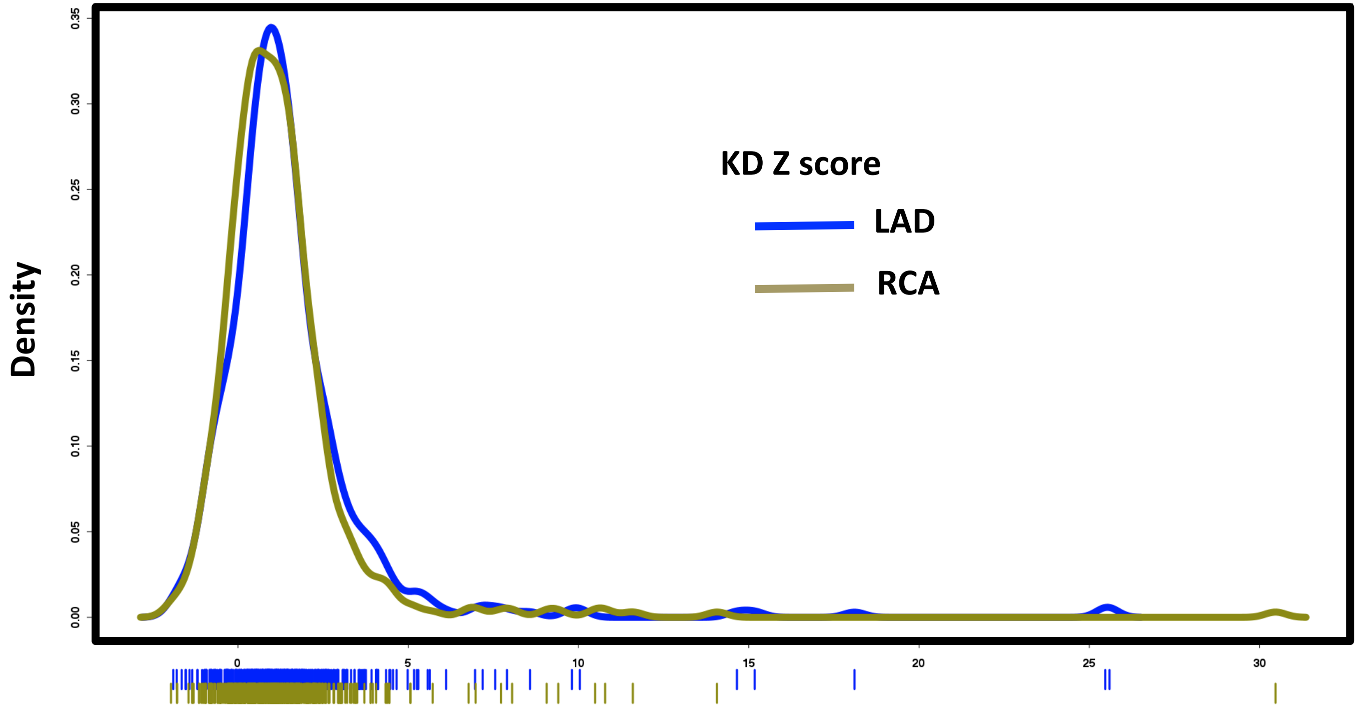

Supplementary Table 1. Differentially expressed genes revealed by csSAM analysis of whole blood expression data set.
NSC value: nearest shrunken centroid value. 1-score: KD patient NSC value; 2-score: FC patient NSC value.

ID	genename	NSC value	
		1-score	2-score
1	TLR7	-0.5752	0.8268
2	CXCL10	-0.5694	0.8185
3	LMO2	-0.5613	0.8068
4	PLXDC1	-0.5604	0.8056
5	MARCH1	-0.539	0.7748
6	IFI30	-0.5308	0.763
7	LYN	-0.5264	0.7567
8	CDC42EP2	-0.5247	0.7542
9	MS4A14	-0.5239	0.7531
10	PARP14	-0.5189	0.746
11	RAC2	-0.5167	0.7428
12	SRF	-0.496	0.713
13	NKTR	-0.494	0.7101
14	LAP3	-0.4904	0.7049
15	APOL3	-0.4791	0.6887
16	STAT1	-0.4718	0.6782
17	GCNT1	-0.4667	0.6709
18	CAMK4	-0.4633	0.666
19	STAT1	-0.4471	0.6427
20	CAMK4	-0.4421	0.6356
21	MRPS25	-0.4169	0.5993
22	P2RY8	-0.4086	0.5874
23	ADD3	-0.3915	0.5629
24	TRIM26	-0.3915	0.5628
25	ARRB1	-0.3761	0.5406
26	GNAS	-0.3676	0.5285
27	ISG20	-0.3635	0.5226
28	PCGF5	-0.3538	0.5086
29	PRPF18	-0.3506	0.504
30	CRTAM	-0.3478	0.4999
31	LHPP	-0.3438	0.4942
32	RASGRP1	-0.3393	0.4877
33	CMPK2	-0.3372	0.4847
34	MS4A14	-0.3345	0.4808
35	TLR7	-0.3341	0.4803
36	RHOH	-0.3328	0.4784
37	DTX4	-0.3024	0.4347
38	SACM1L	-0.2952	0.4244
39	TLR7	-0.2941	0.4227
40	JOSD3	-0.2917	0.4194
41	ARHGAP26	-0.2846	0.4091
42	STAT1	-0.2839	0.4082
43	NBN	-0.2825	0.4061
44	TTN	-0.279	0.4011
45	SKP1	-0.2733	0.3929
46	PEA15	-0.2716	0.3904
47	ZFP106	-0.262	0.3766
48	SEZ6L	-0.2595	0.373
49	CIB1	-0.2512	0.3611
50	HIST1H4C	-0.2489	0.3579
51	KCNJ1	-0.2486	0.3574
52	LTA4H	-0.2405	0.3457
53	TRIM56	-0.2383	0.3426
54	PLCB1	-0.2191	0.315
55	ABCC1	-0.2162	0.3108
56	PTPRCAP	-0.2135	0.3069
57	CCL5	-0.2095	0.3012
58	VAV1	-0.1987	0.2856
59	RBM15	-0.1956	0.2812
60	LOC23117	-0.1878	0.2699
61	MALAT1	-0.1873	0.2692
62	HCCS	-0.1545	0.222
63	C4orf41	-0.1465	0.2105
64	ISG20	-0.1418	0.2039
65	UCN	-0.133	0.1912
66	TP53BP1	-0.1318	0.1895
67	NCAN	-0.1299	0.1867
68	STAM2	-0.1253	0.1801
69	MRPL30	-0.1235	0.1775
70	CCNT1	-0.1234	0.1774
71	C11orf30	-0.1231	0.177
72	POLR1C	-0.1229	0.1766
73	CCND2	-0.1213	0.1744
74	C1QTNF5	-0.1205	0.1732
75	HILS1	-0.1184	0.1703
76	TNFRSF1A	-0.1179	0.1695
77	PRKACA	-0.1148	0.1651
78	NPAT	-0.1125	0.1617
79	MTERF	-0.1058	0.1522
80	ATG16L2	-0.1029	0.148
81	SLAIN1	-0.0935	0.1343
82	IL6ST	-0.0918	0.1319
83	MRAS	-0.0911	0.1309
84	NOTCH4	-0.0864	0.1242
85	ZNF107	-0.0834	0.1199
86	THYN1	-0.0819	0.1177
87	NCOA2	-0.081	0.1164
88	TACC1	-0.0749	0.1077
89	PI4K2B	-0.0716	0.1029
90	DMXL1	-0.0711	0.1022
91	HSPH1	-0.066	0.0949
92	C9orf78	-0.0631	0.0907
93	INTS9	-0.0614	0.0882
94	CYP2J2	-0.0534	0.0767

Supplementary Table 2. Urine peptide biomarker candidates identified by MUDPIT MSMS analysis where spectral count differences between pooled KD and FC samples were greater than 2. m/z: mass to charge ratio.

M/Z	Peptide ID:Gene Name: Sequence	KD Urine Proteome MUDPIT salt GRADIENT 0mM	50mM	100mM	500mM	1000mM	2500mM	5000mM	sum (SC)	FC Urine Proteome MUDPIT salt Gradient 0mM	50mM	100mM	500mM	1000mM	2500mM	5000mM	sum (SC)	Δ(SC) between KD & FC
956.47	Q7Z333:SETX:D.PLGGGDP*TAR.H	2	1	0	0	0	0	0	3	0	0	0	0	0	0	0	0	3
1055.51	P39060:COL18A1:A.GPP*GPP*GPPGPP.G	0	1	1	0	0	0	0	2	0	0	0	0	0	0	0	0	2
1070.49	P20849:COL9A1:G.PP*GP*PGLPGFC.E	0	0	0	0	0	0	0	0	0	2	0	0	0	0	0	2	-2
1101.51	P39060:COL18A1:G.PP*GPP*GPP*GPPS.F	0	0	0	0	0	0	0	0	0	2	0	0	0	0	0	2	-2
1101.55	Q14031:COL4A6:G.LPGPP*GPP*GPPS.P	1	1	0	0	0	0	0	2	0	0	0	0	0	0	0	0	2
1121.47	Q96A84:EMID1:P.P*GPP*GP*MGPPGP*.P	0	0	0	0	0	0	0	0	1	1	0	0	0	0	0	2	-2
1142.58	Q14031:COL4A6:D.GLPGPP*GPPGPPS.P	0	4	0	0	0	0	0	4	0	0	0	0	0	0	0	0	4
1144.55	Q03692:COL1:P.GLPGPP*GPP*GPP*G.Q	10	2	0	0	0	0	0	12	0	1	0	0	0	0	0	1	11
1158.53	P39060:COL18A1:Q.GPP*GPP*GPP*GPPS.F	18	1	0	0	0	0	0	19	0	2	0	0	0	0	0	2	17
1159.52	Q92838:EDA:N.GPP*GPP*GPP*GPQG.P	2	0	0	0	0	0	0	2	0	0	0	0	0	0	0	0	2
1161.58	A6NNG4:COL4A4:P.GP*PGPIGDPGPKG.F	2	0	0	0	0	0	0	2	0	0	0	0	0	0	0	0	2
1175.55	Q14993:COL19A1:P.GPP*GPP*GPP*GIQG.I	3	0	0	0	0	0	0	3	0	1	0	0	0	0	0	1	2
1183.56	B1A8Z6:SMARCA4:G.P*GPGPGP*GPGPAPP.N	0	0	0	0	0	0	0	0	2	0	0	0	0	0	0	2	-2
1218.61	A6NK82:COL4A1:L.PGLP*GPMGP*PGLP.G	2	0	0	0	0	0	0	2	0	0	0	0	0	0	0	0	2
1220.59	P20849:COL9A1:D.GDRGPKGPP*GPP*G.P	0	0	0	0	0	0	0	0	2	0	0	0	0	0	0	2	-2
1224.56	P20849:COL9A1:V.PGPP*GP*PGLPGFC.E	0	1	0	0	0	0	0	1	0	1	2	0	0	0	0	3	-2
1235.60	Q70EL4:USP43:S.RP*GDSPPRPQP*.G	0	0	0	0	0	0	0	0	4	0	0	0	0	0	0	4	-4
1236.58	Q15464:SH:L.GVGSGRP*GGP*PPGP*.G	0	2	0	0	0	0	0	2	0	0	0	0	0	0	0	0	2
1251.55	P02452:COL1A1:G.AP*GDRGEP*GPP*GP.A	5	3	0	0	0	0	0	8	0	1	0	0	0	0	0	1	7
1263.77	P49750:YLPM:S.LPPLPPLPPLP*P.L	0	0	0	0	0	0	0	0	0	1	0	0	0	1	0	2	-2
1310.52	Q01851:POU4F1:D.GP*GGGGGPGGGGGP*GGGGP*.G	0	0	0	0	0	0	0	0	0	0	1	0	1	0	0	2	-2
1315.62	P02452:COL1A1:G.SP*GAKGDRGETGPA.G	0	1	1	0	0	0	0	2	0	0	0	0	0	0	0	0	2
1324.76	P07911:UMOD:V.IDQSRVLNLGPI.T	0	1	1	0	0	0	0	2	0	0	0	0	0	0	0	0	2
1338.61	P02452:COL1A1:V.GPP*GPP*GPP*GPP*GPP.S	0	0	4	1	0	0	0	5	0	0	2	0	0	0	0	2	3
1339.62	P20849:COL9A1:G.VP*GP*PGP*PGLPGFC.E	0	0	1	2	0	0	0	3	0	0	0	0	0	0	0	0	3
1352.63	P02452:COL1A1:G.P*PGPP*GPPGP*PGPPS.A	0	0	1	2	0	0	0	3	0	0	0	0	0	0	0	0	3
1353.80	P07911:UMOD:D.QSRVLNLGPITR.K	0	0	0	0	0	0	0	0	0	0	0	1	1	0	0	2	-2
1368.62	Q96A84:EMID1:P.P*GPPGP*PGP*PGP*PAP*.V	0	6	0	0	0	0	0	6	0	3	1	0	0	0	0	4	2
1368.66	P39060:COL18A1:G.PP*GPP*GPP*GPPGTPV.Y	0	0	0	0	0	0	0	0	0	2	0	0	0	0	0	2	-2
1377.81	Q8N8S7:ENAH:I.VLGPLAPPPP*PPLP.P	0	0	1	1	0	0	0	2	0	0	0	0	0	0	0	0	2
1393.66	P02452:COL1A1:V.GPP*GPP*GPPGPPGPPS.A	0	0	0	0	0	0	1	1	0	0	0	0	0	4	0	4	-3
1409.65	Q96A84:EMID1:R.GPP*GPP*GPP*GPPGPP*A.P	0	0	3	0	14	0	0	17	0	0	0	0	0	0	0	0	17
1410.73	B2ZZ86:COL5A1:I.GPP*GPP*GLPGPPGPKG.A	0	0	1	0	2	0	0	3	0	0	0	0	0	0	0	0	3
1410.84	Q9HCN6:GP6:R.AVQRPLPP*LPPLP.Q	0	0	1	1	0	0	0	2	0	0	0	0	0	0	0	0	2
1414.68	P12107:COL11A1:V.GPMGPPGPPGP*RGPQ.G	0	0	2	0	0	0	0	2	0	0	0	0	0	0	0	0	2
1424.65	P81408:C1orf2:S.GPLGPLGCTSP*P*P*AP*.L	0	0	0	0	0	0	0	0	0	1	1	0	0	0	0	2	-2
1425.64	Q96A84:EMID1:R.GPP*GPP*GPP*GPP*GPP*A.P	0	0	14	0	0	0	0	14	0	0	0	0	0	0	0	0	14
1426.70	P39060:COL18A1:G.RQGPPGPP*GPP*GPPS.F	0	0	0	5	3	0	0	8	0	0	0	0	0	0	0	0	8
1426.72	Q14050:COL9A3:V.GLPGPP*GPP*GPP*GKPG.Q	0	1	4	3	1	0	0	9	0	0	0	0	0	0	0	0	9
1427.62	Q9NR64:KLHL1:K.WNMCAP*M#CKRR.G	0	0	0	0	0	0	0	0	0	2	0	0	0	0	0	2	-2
1427.67	Q03692:COL1:S.GEP*GLPGP*PGP*PGPPG.Q	0	0	0	2	0	1	0	3	0	0	0	0	0	0	0	0	3
1427.69	Q99715:COL12A1:R.GDIGPPGPQGPPGPQG.P	0	0	0	1	1	0	0	2	0	0	0	0	0	0	0	0	2
1427.73	P08572:COL4A2:V.GAPGRPGPP*GPP*GPKG.Q	0	0	1	0	1	0	0	2	0	0	0	0	0	0	0	0	2
1442.69	P39060:COL18A1:G.RQGPP*GPP*GPP*GPPS.F	0	0	1	1	0	0	0	2	0	0	0	0	0	0	0	0	2
1442.71	Q14050:COL9A3:V.GLPGPP*GPP*GPP*GKP*G.Q	1	9	0	0	0	0	0	10	0	6	0	0	0	0	0	6	4
1442.72	Q9UMD9:COL17A1:S.GVQGPP*GPPGPP*GPVT.T	0	1	2	0	0	0	0	3	0	0	0	0	0	0	0	0	3
1445.75	A6NK82:COL4A1:P.GLPGLPGPM#GPP*GLPG.I	3	0	0	0	0	0	0	3	0	0	0	0	0	0	0	0	3
1446.67	Q6AZY7:SCARA3:Q.GPP*GLPGP*PGPP*GSQS.F	0	0	2	0	0	0	0	2	0	0	0	0	0	0	0	0	2
1449.70	Q5TAT6:COL13A1:P.PGP*PGPPGPSGPLGHP*.G	0	0	0	0	0	0	0	0	0	2	0	0	0	0	0	2	-2
1492.70	P39059:COL15A1:R.PGDPGPPGPP*GPPGPP*A.I	0	0	0	0	0	1	0	1	0	0	0	0	2	1	0	3	-2
1505.78	Q96A83:EMID2:A.GVPGP*RGPPGPPGPPGP.R	0	0	0	0	0	0	0	0	0	0	0	2	0	0	0	2	-2
1508.72	P02452:COL1A1:P.VGP*PGPP*GPP*GPPGPPS.A	0	0	0	1	2	0	0	3	0	0	0	0	0	0	0	0	3
1509.69	P02461:COL3A1:A.GPP*GM#P*GPRGSP*GPQG.V	0	0	0	0	0	0	0	0	0	0	0	0	1	1	0	2	-2
1509.78	Q86Y22:COL23A1:Q.LIVEPGP*PGP*PGPPGP.M	0	0	0	1	6	1	0	8	0	0	1	2	0	0	0	3	5
1510.86	P07911:UMOD:G.SVIDQSRVLNLGPI.T	0	1	1	0	0	0	0	2	0	0	0	0	0	0	0	0	2
1523.69	XP 001713935:FLJ34208:S.TAEDELGSRTDSSR.A	0	0	0	0	1	0	1	2	0	0	0	0	0	0	0	0	2
1524.71	P02452:COL1A1:P.VGPP*GPP*GPP*GPP*GPPS.A	0	21	23	1	0	0	0	45	1	22	25	0	0	0	0	48	-3
1525.76	P02452:COL1A1:R.DGIPGQPGLP*GPPGPPG.P	0	0	0	1	1	0	0	2	0	0	0	0	0	0	0	0	2
1525.77	Q86Y22:COL23A1:Q.LIVEP*GP*PGP*PGPPGP.M	0	3	2	0	0	0	0	5	0	0	0	0	0	0	0	0	5
1526.75	Q9UMD9:COL17A1:F.PGTP*GIPGP*LGHP*GPQ.G	0	0	0	2	0	0	0	2	0	0	0	0	0	0	0	0	2
1528.73	OTTHUMP00000194899:COL16A1:S.GMP*GPP*GIPGP*PGPPGV.P	0	0	0	0	0	0	0	0	0	1	0	0	0	1	0	2	-2
1530.74	Q99715:COL12A1:S.VGPP*GPP*GP*AGGPGAKGP*.R	0	0	0	2	0	0	0	2	0	0	0	0	0	0	0	0	2
1541.75	Q5KU26:COLEC12:S.GDPGPP*GPPGKEGLPGP.Q	0	0	2	0	0	0	0	2	0	0	0	0	0	0	0	0	2
1541.76	Q99700:ATXN2:P.SAAPPPP*GPGP*PPSRQ.S	0	0	0	0	0	0	0	0	0	1	1	0	0	0	0	2	-2
1542.74	Q14031:COL4A6:G.RDGLPGP*PGP*PGP*PSP.E	0	0	2	0	0	0	0	2	0	0	0	0	0	0	0	0	2
1557.77	P02452:COL1A1:A.AGRVGPP*GPSGNAGPPGP.P	0	7	0	0	0	0	0	7	0	3	0	0	0	0	0	3	4
1563.77	P02452:COL1A1:P.VGPP*GPP*GPPGPPGPPSA.G	0	0	0	0	0	0	0	0	0	0	0	0	0	0	2	2	-2
1567.74	Q96A84:EMID1:P.GERGP*PGPP*GP*PGPPGP.P	0	0	0	0	0	0	0	0	0	1	2	0	0	0	0	3	-3
1579.81	HIT000009311:-:H.VPVQTWNPDGLQIP*.S	0	0	2	0	0	0	0	2	0	0	0	0	0	0	0	0	2
1581.91	P07911:UMO:V.IDQSRVLNLGPITR.K	0	0	0	3	3	1	0	7	0	0	0	1	2	0	0	3	4
1590.79	P02452:COL1A1:G.DRGETGPAGPAGPVGPVG.A	0	0	1	1	0	0	0	2	0	0	0	0	0	0	0	0	2
1600.67	Q8WXR4:MYO3B:A.GDTSNQSSGP*HSP*VAAG.T	0	0	1	0	0	1	0	2	0	0	0	0	0	0	0	0	2
1611.91	P07911:UMO:G.SVIDQSRVLNLGPIT.R	0	1	0	1	0	0	0	2	0	0	0	0	0	0	0	0	2
1635.87	P58505:C21orf58:P.SRGALGSALP*PELPPTG.I	0	0	1	1	0	0	0	2	0	0	0	0	0	0	0	0	2
1637.85	Q14050:COL9A3:R.VGLPGPP*GPP*GPPGKPGQ.D	0	0	0	0	4	0	0	4	0	0	0	0	0	0	0	0	4
1644.79	P25940:C:A.PGAKGNVGPP*GEPGPP*GQ.Q	0	0	0	0	0	0	0	0	0	0	0	1	1	0	0	2	-2
1649.83	Q8IWN7:RP1L1:K.P*P*KTPSGPGRPQERN.P	0	0	0	0	0	0	0	0	0	0	0	1	1	0	0	2	-2
1652.79	Q96A84:EMID1:G.TPGERGPP*GPP*GPPGPPG.P	0	0	7	0	0	0	0	7	0	0	15	1	0	0	0	16	-9
1653.70	Q6PID8:KLHDC10:N.TNLYVFGGYNP*DYD.E	0	1	0	1	0	0	0	2	0	0	0	0	0	0	0	0	2
1653.77	Q6ZMI3::A.KGDQGP*PGP*PGP*PGPPGP.P	0	1	6	1	0	0	0	8	0	1	1	0	0	0	0	2	6
1653.84	Q14050:COL9A3:R.VGLPGPP*GPP*GPP*GKPGQ.D	0	0	5	4	4	0	0	13	0	0	3	0	0	0	0	3	10
1654.81	Q02388:NA:G.RDGP*PGLPGTP*GPPGPPG.P	0	0	0	0	4	4	0	8	0	0	0	0	0	0	0	0	8
1669.83	Q14050:COL9A3:R.VGLPGPP*GPP*GPP*GKP*GQ.D	0	0	7	0	1	0	0	8	0	0	1	0	0	0	0	1	7
1670.78	P20849:COL9A1:P.P*GEQGP*PGP*PGP*PGVPGI.D	0	0	3	1	0	0	0	4	0	0	0	0	0	0	0	0	4
1696.75	Q9H6T0:ESRP2:M.TPPPP*PP*P*PP*GPDP*AAD.P	1	0	1	1	0	0	0	3	0	0	0	0	0	0	0	0	3
1717.78	P39060:COL18A1:M.P*GPPGP*PGP*PGP*PGTP*VY.D	0	0	0	0	0	0	0	0	0	6	0	0	0	0	0	6	-6
1742.83	Q96A84:EMID1:P.GP*PGPP*GPP*GPPAPVGPP*H.A	2	0	0	0	0	0	0	2	0	0	0	0	0	0	0	0	2
1755.96	P07911:UMOD:R.SGSVIDQSRVLNLGPIT.R	0	1	0	0	0	0	0	1	0	1	1	1	0	0	0	3	-2
1768.01	P07911:UMOD:G.SVIDQSRVLNLGPITR.K	0	0	0	0	1	1	0	2	0	0	0	0	0	0	0	0	2
1768.99	Q9HCF6:TRPM3:L.KLLGMEDDIPLRRGR.K	0	0	0	0	0	0	0	0	0	0	0	0	1	1	0	2	-2
1773.91	P39060:COL18A1:D.GVGQPGLPGPP*GPP*GPVVY.V	0	0	0	0	0	0	0	0	0	1	0	0	1	0	0	2	-2
1824.86	Q1KMD3:HNRNPUL2:D.EAGGGGAGP*GGACKAEPRPVA.A	0	0	0	0	0	0	0	0	0	1	1	0	0	0	0	2	-2
1825.03	P07911:UMOD:S.GSVIDQSRVLNLGPITR.K	0	0	0	1	0	1	0	2	0	0	0	0	0	0	0	0	2
1848.86	P02452:COL1A1:G.DAGPVGPP*GPP*GPPGP*PGPPS.A	0	0	0	0	0	0	0	0	0	2	0	0	0	0	0	2	-2
1850.91	P39060:COL18A1:M.RGMPGPP*GPP*GPPGPPGTPV.Y	0	2	3	0	0	0	0	5	0	3	0	0	0	0	0	3	2
1850.93	Q14031:COL4A6:A.GLPGRDGLPGP*PGP*PGPPSP.E	0	0	2	0	0	0	0	2	0	0	0	0	0	0	0	0	2
1864.86	Q96A84:EMID1:T.PGERGPP*GPP*GPP*GPP*GPP*A.P	0	3	0	0	0	0	0	3	0	6	0	0	0	0	0	6	-3
1866.90	P39060:COL18A1:M.RGMP*GPP*GPP*GPPGPPGTPV.Y	0	1	1	0	0	0	0	2	0	0	0	0	0	0	0	0	2
1870.86	Q9HBU9:POPDC2;COX17:L.QQTPPCSTPPATTNFPAP*.P	0	0	0	0	0	0	0	0	0	0	2	0	0	0	0	2	-2
1877.86	Q9UMD9:COL17A1:S.SMLTVP*GPP*GPP*GAMGPP*GP.P	0	2	0	0	0	0	0	2	0	0	0	0	0	0	0	0	2
1882.89	P39060:COL18A1:M.RGMP*GPP*GPP*GPP*GPPGTPV.Y	0	3	0	0	0	0	0	3	0	0	0	0	0	0	0	0	3
1914.03	P48995:TRPC1:D.KCTLPP*P*FNIIP*SPKTI.C	0	0	0	1	0	1	0	2	0	0	0	0	0	0	0	0	2
1928.89	Q86Y22:COL23A1:A.GPP*GPP*GP*PGARGPPGDTGKD.G	0	0	0	0	2	0	0	2	0	0	0	0	0	0	0	0	2
1972.95	P67809:YBX1:D.TKPGTTGSGAGSGGP*GGLTSAAPA.G	0	1	1	0	0	0	0	2	0	0	0	0	0	0	0	0	2
1987.84	P98174:FGD1:P.EHPATNPPGAAPPACADSDP*G.A	0	0	0	0	0	0	0	0	0	0	2	0	0	0	0	2	-2
2009.96	P39059:COL15A1:K.KGQAGPP*GVMGPP*GPP*GPP*GPP.G	0	0	1	1	0	0	0	2	0	0	0	0	0	0	0	0	2
2029.97	P39060:COL18A1:M.RGMP*GPPGP*PGPPGPPGTP*VY.D	0	0	1	1	0	0	0	2	0	0	0	0	0	0	0	0	2
2032.98	Q07092:CO:P.MGQQGIPGIPGPP*GP*MGQPGK.A	0	0	3	0	0	0	0	3	0	0	0	0	0	0	0	0	3
2032.99	Q07092:CO:P.MGQQGIPGIPGPPGP*M#GQPGK.A	0	0	0	0	3	0	0	3	0	0	0	0	0	0	0	0	3
2033.04	Q9BXS0:COL25A1:R.ITTLTVTGP*PGPP*GP*QGLQGP.K	0	0	1	0	1	0	0	2	0	0	0	0	0	0	0	0	2
2091.02	P0C7U0:ELFN1:G.SHPAEPP*APPGPPPPPPHEGL.G	0	0	0	0	0	2	0	2	0	0	0	0	0	0	0	0	2
2114.98	Q5TAT6:COL13A1:R.GEP*GPP*GLP*GPPGPKGEAGVDGQ.V	0	0	0	1	1	0	0	2	0	0	0	0	0	0	0	0	2
2138.03	P21675:TAF1:E.DIDCKLM#PPPPP*P*PGP*M#KK.D	0	0	2	0	0	0	0	2	0	0	0	0	0	0	0	0	2
2142.05	A6NFA2:COL9A1:R.GP*PGVAGIPGVPGP*PGPP*GLPGFC.E	0	0	1	1	0	0	0	2	0	0	0	0	0	0	0	0	2
2154.16	OTTHUMP00000077011:-:L.AASHSNLLLGP*GGPPTPLRGLP*.P	0	0	1	1	0	0	0	2	0	0	0	0	0	0	0	0	2
2160.03	Q6ZSL6:-:S.PEGGGGAGARGGAWPTAP*AP*LP*PA.T	0	0	0	0	0	0	0	0	0	2	0	0	0	0	0	2	-2
2162.03	Q96A84:EMID1:S.RGPM#GMRGPP*GPQGPP*GSPGRA.G	0	0	0	0	0	0	0	0	0	3	0	0	0	0	0	3	-3
2170.93	Q6E0U4:DMKN:G.MNPQGAPWGQGGNGGP*PNFGTN.T	0	0	0	0	0	0	0	0	0	4	0	0	0	0	0	4	-4
2177.08	P12107:COL11A1:I.PGPAGPLGP*PGP*PGLP*GP*QGPKGN.K	0	0	2	0	0	0	0	2	0	0	0	0	0	0	0	0	2
2187.27	O43711:TLX3:V.IRVP*AHRP*LPGAVPPPLPSAL.P	0	0	2	0	0	0	0	2	0	0	0	0	0	0	0	0	2
2189.95	Q6ZWB6:KCTD8:S.SSSSPGASAAAAP*GP*CAPSPFP*EV.V	0	0	1	0	0	1	0	2	0	0	0	0	0	0	0	0	2
2217.03	P02452:COL1A1:P.IGPP*GPAGAP*GDKGESGPSGPAGPTG.A	0	0	0	0	1	1	0	2	0	0	0	0	0	0	0	0	2
2233.02	P02452:COL1A1:P.IGPP*GPAGAP*GDKGESGP*SGPAGPTG.A	0	0	0	0	1	1	0	2	0	0	0	0	0	0	0	0	2
2252.07	Q9P203:BTBD7:M.FTDLDSFVAFHP*PLPP*PPP*P*.Y	0	0	0	0	0	0	0	0	0	2	0	0	0	0	0	2	-2
2297.37	Q9UBB6:NA:G.RLLLAANVATLGLLMARLLSTS.P	0	0	0	0	0	0	0	0	0	2	0	0	0	0	0	2	-2
2406.15	Q6UWR5:PNKD:S.PQRPP*PPGRQPLCP*SP*PGYLCT.L	0	0	0	0	0	0	0	0	0	2	0	0	0	0	0	2	-2
2436.13	Q05519:SFRS11:G.PGPSGGPGGGGGGGGGGGGTEVIQVTNVSP*.S	0	0	0	0	0	0	0	0	4	0	0	0	0	0	0	4	-4
2450.19	Q9UP45:COL13A1:L.PGPMGPPGLPGPPGPKGDP*GIQGYHG.R	0	0	0	0	0	0	0	0	0	2	0	0	0	0	0	2	-2
2452.22	Q9UPU6:-:L.PPGLMGPGPPPP*PGATPTSPPELFQ.Y	0	0	0	0	0	0	0	0	0	2	0	0	0	0	0	2	-2
2457.25	Q8NFW1:COL22A1:N.DCPPGPP*GLPGLPGFKGDKGVPGKPG.R	0	0	0	0	3	0	0	3	0	0	0	0	0	0	0	0	3
2472.15	P02452:COL1A1:L.TGPIGP*PGPAGAP*GDKGESGPSGPAGPTG.A	0	0	0	0	0	1	1	2	0	0	0	0	0	0	0	0	2
2577.20	Q14C86:GAPVD1:T.ATLHEPIM#QLLVEDEDHLETDP*.N	0	0	0	0	0	0	0	0	0	2	0	0	0	0	0	2	-2
2614.26	P10275:AR:G.LGVEALEHLSP*GEQLRGDCMYAPL.L	0	0	2	0	0	0	0	2	0	0	0	0	0	0	0	0	2
2681.25	Q5T124:UBXN11:S.SLKFSPGP*CPGPGPGPSPGPGP*GPSPGP*GP.G	0	0	0	0	0	1	1	2	0	0	0	0	0	0	0	0	2
2691.37	P01011:SERPINA3:R.FNRP*FLMIIVPTDTQNIFFM#SK.V	0	0	0	0	0	0	2	2	0	0	0	0	0	0	0	0	2
2691.65	P51787:KCNQ1:Y.VGLWGRLRFARKPISIIDLIVVVA.S	0	0	0	2	0	0	0	2	0	0	0	0	0	0	0	0	2
2691.66	Q4AC86:MEGF6:V.VLALVLLLLP*AVP*VGASVP*PRP*LLPL.Q	0	0	0	1	0	0	1	2	0	0	0	0	0	0	0	0	2
2692.64	O15439:ABCC4:V.VSVAVAVIP*WIAIP*LVPLGIIFIFL.R	0	0	0	1	0	0	1	2	0	0	0	0	0	0	0	0	2
2819.32	P39060:COL18A1:P.GPPGPQGP*P*GIGYEGRQGPP*GPP*GPPGPPS.F	0	0	0	1	0	1	0	2	0	0	0	0	0	0	0	0	2

Demographics of the 46 Patients with Peptidome Data Analyzed by FTICR MUDPIT

	**Kawasaki Disease**	**Febrile Condition**	**p-value**
	n = 23 (50.0%)	n = 23 (50.0%)	
Age (months)^	37.6 [3.0, 94.0, 17.4-32.3]	47.6 [8.0, 191.0, 35.9-65.8]	0.361
Male	17 (73.9%)	14 (60.8%)	0.189
Ethnicity			
Asian	3 (13.6%)	4 (17.4%)	0.866
African American	0 (0%)	1 (4.4%)	
Caucasian	6 (27.3%)	4 (17.4%)	
Hispanic	6 (27.3%)	8 (34.8%)	
Mixed	7 (31.8%)	6 (26.1%)	

^ Reported as means with minimum, maximum, and 95% confidence interval in bracket. T-test was used.
All other variables were reported as the number of patients and were analyzed using Fisher's Exact test.

Supplementary Table 3

Demographics of the 60 Patients with Peptidome Data Profiled by MALDI 5800

	**Kawasaki Disease**	**Febrile Condition**	**p-value**
	n = 30 (50.0%)	n = 30 (50.0%)	
Age (months)^	60.0 [5.0, 182.0, 42.3-77.7]	59.3 [5.0, 209.0, 41.1-77.4]	0.951
Male	19 (63.3%)	19 (63.3%)	1.000
Ethnicity			
Asian	4 (13.3%)	2 (6.7%)	0.025
African American	1 (3.3%)	0 (0%)	
Caucasian	4 (13.3%)	8 (26.7%)	
Hispanic	12 (40.0%)	8 (26.7%)	
Mixed	9 (30.0%)	5 (16.7%)	

^ Reported as means with minimum, maximum, and 95% confidence interval in bracket. T-test was used.
All other variables were reported as number of patients and analyzed using Fisher's Exact test.

Supplementary Table 4
